# Biased Perception of Physiological Arousal in Child Social Anxiety Disorder Before and After Cognitive Behavioral Treatment

**DOI:** 10.32872/cpe.v2i2.2691

**Published:** 2020-06-30

**Authors:** Julia Asbrand, André Schulz, Nina Heinrichs, Brunna Tuschen-Caffier

**Affiliations:** a Institute of Psychology, Albert Ludwigs University of Freiburg, Freiburg, Germany; b Institute of Psychology, Humboldt-Universität zu Berlin, Berlin, Germany; c Clinical Psychophysiology Laboratory, Institute for Health and Behaviour, University of Luxembourg, Esch-sur-Alzette, Luxembourg; d Department of Psychology, University of Bremen, Bremen, Germany; Philipps-University of Marburg, Marburg, Germany

**Keywords:** bodily arousal, social phobia, CBT, therapy, interoceptive awareness, heartbeat perception

## Abstract

**Background:**

A biased perception of physiological hyperreactivity to social-evaluative situations is crucial for the maintenance of social anxiety disorder (SAD). Alterations in interoceptive accuracy (IAc) when confronted with social stressors may play a role for SAD in children. We expected a biased perception of hyperarousal in children with SAD before treatment and, consequently, a reduced bias after successful cognitive behavioral therapy (CBT).

**Method:**

In two centers, 64 children with the diagnosis of SAD and 55 healthy control (HC) children (both 9 to 13 years) participated in the Trier Social Stress Test for Children (TSST-C), which was repeated after children with SAD were assigned to either a 12-week group CBT (n = 31) or a waitlist condition (n = 33). Perception of and worry about physiological arousal and autonomic variables (heart rate, skin conductance) were assessed. After each TSST-C, all children further completed a heartbeat perception task to assess IAc.

**Results:**

Before treatment, children with SAD reported both a stronger perception of and more worry about their heart rate and skin conductance than HC children, while the objective reactivity of heart rate did not differ. Additionally, children with SAD reported heightened perception of and increased worry about trembling throughout the TSST-C compared to HC children, but reported increased worry about blushing only after the stress phase of the TSST-C compared to HC children. Children with and without SAD did not differ in IAc. Contrary to our hypothesis, after treatment, children in the CBT group reported heightened perception of physiological arousal and increased worry on some parameters after the baseline phase of the TSST-C, whereas actual IAc remained unaffected. IAc before and after treatment were significantly related.

**Conclusions:**

Increased self-reported perception of physiological arousal may play a role in childhood SAD and could be an important target in CBT. However, further studies should examine if this is an epiphenomenon, a temporarily occurring and necessary condition for change, or indeed an unwanted adverse intervention effect.

## Abstract (highlights)

- Biased perception of physiological arousal may play a role in child social anxiety disorder (SAD).
- Faced with standardized social stress, biased perception of heart rate but not skin conductance.
- No change in biased perception due to cognitive-behavioral treatment.
- Further research regarding the nature of biased perception (e.g. epiphenomenon) necessary.

Social anxiety disorder (SAD) is a highly prevalent disorder (Burstein et al., 2011) that leads to great impairment in the well-being and everyday life of affected children (Rao et al., 2007). Cognitive models of SAD (e.g., Clark & Wells, 1995) point to the importance of an increased focus on cognitions, feelings, and behaviors. In addition, a person with SAD is also alarmed by physiological reactions in social situations. In line with cognitive models, the subjective awareness of physiological and emotional arousal is interpreted negatively, which leads to an overall negative self-perception followed by elevated fear of and avoidance of social situations.

A (physiological) anxiety reaction was required in the Diagnostic and Statistical Manual for Mental Disorders (4th ed., text rev.; DSM-IV-TR; American Psychiatric Association [APA], 2000). This has been revised in the latest version, allowing to display any sign of fear, not necessarily physiologically (DSM-5, APA, 2013). This change reflects that the objective physiological reaction is not yet fully understood: Several studies have shown tonic hyperarousal in children with SAD (Asbrand, Blechert, Nitschke, Tuschen-Caffier, & Schmitz, 2017; Krämer et al., 2012; Miers, Blöte, Sumter, Kallen, & Westenberg, 2011; Schmitz, Tuschen-Caffier, Wilhelm, & Blechert, 2013). However, research has failed to find heightened physiological reactivity to disorder-typical stress (for an overview see Siess, Blechert, & Schmitz, 2014). Still, both children and adults with SAD have reported increased perception of physiological arousal (Gerlach, Mourlane, & Rist, 2004; Schmitz, Blechert, Krämer, Asbrand, & Tuschen-Caffier, 2012). Therefore, it has been hypothesized that cognitive factors (e.g., attention allocation and evaluation) are also relevant for physiological factors. That is, people with SAD are more prone to shift their attention towards physiological arousal and evaluate this arousal as more threatening (Clark & Wells, 1995; Siess et al., 2014). Attentional biases have previously been examined mostly for external cues, such as angry versus happy faces, with measures of reaction times or with eye tracking (for an overview in adults see Bar-Haim, Lamy, Pergamin, Bakermans-Kranenburg, & van Ijzendoorn, 2007). Similar to studies of adults, a meta-analysis of anxious compared to nonanxious children (Dudeney, Sharpe, & Hunt, 2015) showed a significant attentional bias to threat. While these findings on external attentional biases are in line with Rapee and Heimberg's (1997) theoretical model of SAD, the importance of other biases, also suggested by current cognitive models (e.g., Clark & Wells, 1995; Rapee & Heimberg, 1997) have received less attention, specifically internal perceptional biases. The processing of internal perceptional information is likely dependent upon their (believed) visibility for others:

Certain internal symptoms (e.g. increased heart rate, nausea) are relevant for the experience of anxiety in general but are not overly visible (cognition: “My heart is racing, this must mean that I am anxious”). However, other physiological symptoms are clearly visible (e.g. blushing, sweating, trembling) and are, therefore, extremely relevant for the fear of being judged (cognition: “I am blushing, others can see how anxious I am”). As such, these physiological symptoms are relevant for the experience of SAD specifically. One previous study in children aged 10 to 12 years with high versus low social anxiety (Schmitz et al., 2012) manipulated heart rate visibility by applying a heart rate feedback tone while children told a story in a “private” condition (head phones) and a “public” condition (with adult observers present). Children with high social anxiety perceived their heart rate as higher than low socially anxious children when they listened to their (supposedly own) heart rate both in private and in public with adult observers present. Further, the public condition led to more worry about the heart rate visibility only in children with high social anxiety. This study demonstrated that both perception of and worry about visibility of physiological arousal (i.e. evaluation) is elevated in socially anxious children. As this study examined a subclinical sample, it is necessary to assess children with SAD to assure the stability of this phenomenon in clinically affected children. Additionally, as the study used a set-up specific to perception of and worry about heart rate, it should be tested if this finding is stable in a well-established social stress test, the Trier Social Stress Test for Children (TSST-C; Buske-Kirschbaum et al., 1997) and using more than one physiological parameter (Siess et al., 2014).

To reveal the underlying processes of biased perception in children with SAD, it is required to assess different facets of interoception: First, ‘interoceptive accuracy’ (IAc) represents the correspondence between actual and perceived physical signals (e.g., heartbeats). Second, the subjective tendency to be focused on physical signals is considered ‘interoceptive sensibility’ (IS) (Garfinkel, Seth, Barrett, Suzuki, & Critchley, 2015). Third, ‘interoceptive evaluation’ (IE) reflects subjective affective valence of physical sensations such as worry about visibility (Pollatos & Herbert, 2018). While the attentional biases refer more closely to IS and IE, IAc should be additionally considered. The heartbeat counting task (HCT) has been established as most common approach to assess cardiac IAc in adults and in school children with and without anxiety symptoms (Eley, Gregory, Clark, & Ehlers, 2007; Eley, Stirling, Ehlers, Gregory, & Clark, 2004; Georgiou et al., 2015; Koch & Pollatos, 2014; Schandry, 1981; Schmitz et al., 2012). For example, Antony et al. (1995) assessed IAc based on HCT and heart rate (HR) in adult patients with panic disorder and SAD compared to healthy controls (HC). Groups did not differ in IAc at rest or after exercise. However, self-reported anxiety was positively related to IAc. In a child community sample (Eley et al., 2004), children between 8 and 11 years completed the HCT. After a distinction into good and poor heartbeat perceivers based on IAc scores, good perceivers reported significantly higher panic and/or somatic symptoms and were more sensitive to anxiety. Similarly, higher levels of panic and/or somatic symptoms were positively related to IAc. Both findings suggest that a proper perception of physical sensations (IAc) enhances their interpretation as potentially threatening (IE) in SAD. Furthermore, Schmitz et al. (2012) did not find differences in IAc based on the HCT between children with high and low social anxiety, which implies that IAc and IE may dissociate under specific circumstances. The authors assume that socially anxious children overestimate their HR under stress (i.e. over-reporting of cardiac sensations) but are able to perceive their heartbeat correctly in the recovery period after stress (Mauss, Wilhelm, & Gross, 2004; Pollatos, Traut-Mattausch, Schroeder, & Schandry, 2007). In summary, the role of IAc, IS and IE (including over-reporting of cardiac sensations) in fully manifested SAD remains unclear.

If biased perception (IS) and evaluation (IE) of physiological symptoms and/or IAc are central factors in childhood SAD, a longitudinal assessment measuring stability and changeability by treatment is a plausible next step (e.g., Siess et al., 2014). Once again, previous research focused on treatment effects on other biases, for example, interpretation biases (Leigh & Clark, 2018). However, theoretical models placed the misperception of physiological symptoms as central for SAD (e.g., Clark & Wells, 1995), which leads to the assumption that cognitive behavioral therapy (CBT) might change this perception bias. Interestingly though, most treatments of SAD do not explicitly focus on a biased perception of physiological symptoms but rather on general cognitions in and after social situations and on behavior in children (e.g., Beidel & Turner, 2007). However, as pointed out above, the importance of including specific treatment components targeting physiological reactions cannot be fully supported by empirical evidence, as findings on physiological hyperarousal are inconsistent (Siess et al., 2014).

## The Current Study

On objective measures (heart rate, electrodermal activity [EDA]), we expected children with SAD to show only tonic hyperarousal and no increased reactivity to social stress compared to children in a healthy control (HC) group (Asbrand et al., 2017; Schmitz et al., 2013). On subjective measures, we expected all children to report perception (i.e. IS) of and worry (i.e. IE) about physiological variables (heart rate, perspiration, blushing, trembling)^1^1Heart rate and perspiration were chosen to be assessed objectively as well. While the project further included other physiological variables (e.g. cortisol; Asbrand, Heinrichs, Nitschke, Wolf, Schmidtendorf, & Tuschen-Caffier, 2019), these do not have a subjective counterpart which can be assessed by self-report. Due to technical limitations, we could not assess blushing and trembling as objective parameters. that increases from baseline to stress and then decreases to recovery. This effect, that is, heightened perceived reactivity, has been hypothesized to be stronger in children with SAD compared to HC children (Schmitz et al., 2012). In line with previous findings, we expect a positive correlation between physiological activation (e.g., heart rate) and IAc. After children with SAD were assigned to a treatment (group CBT) or waitlist control (WLC) group, we expected only small differences in objective measures (heart rate, EDA) on a second TSST-C. We expected differences in subjective measures, i.e. children in the CBT group reporting less perception of and worry about physiological variables compared to children in the WLC group and compared to results of the TSST-C before treatment.

## Method

### Trial Design

**Figure 1:**
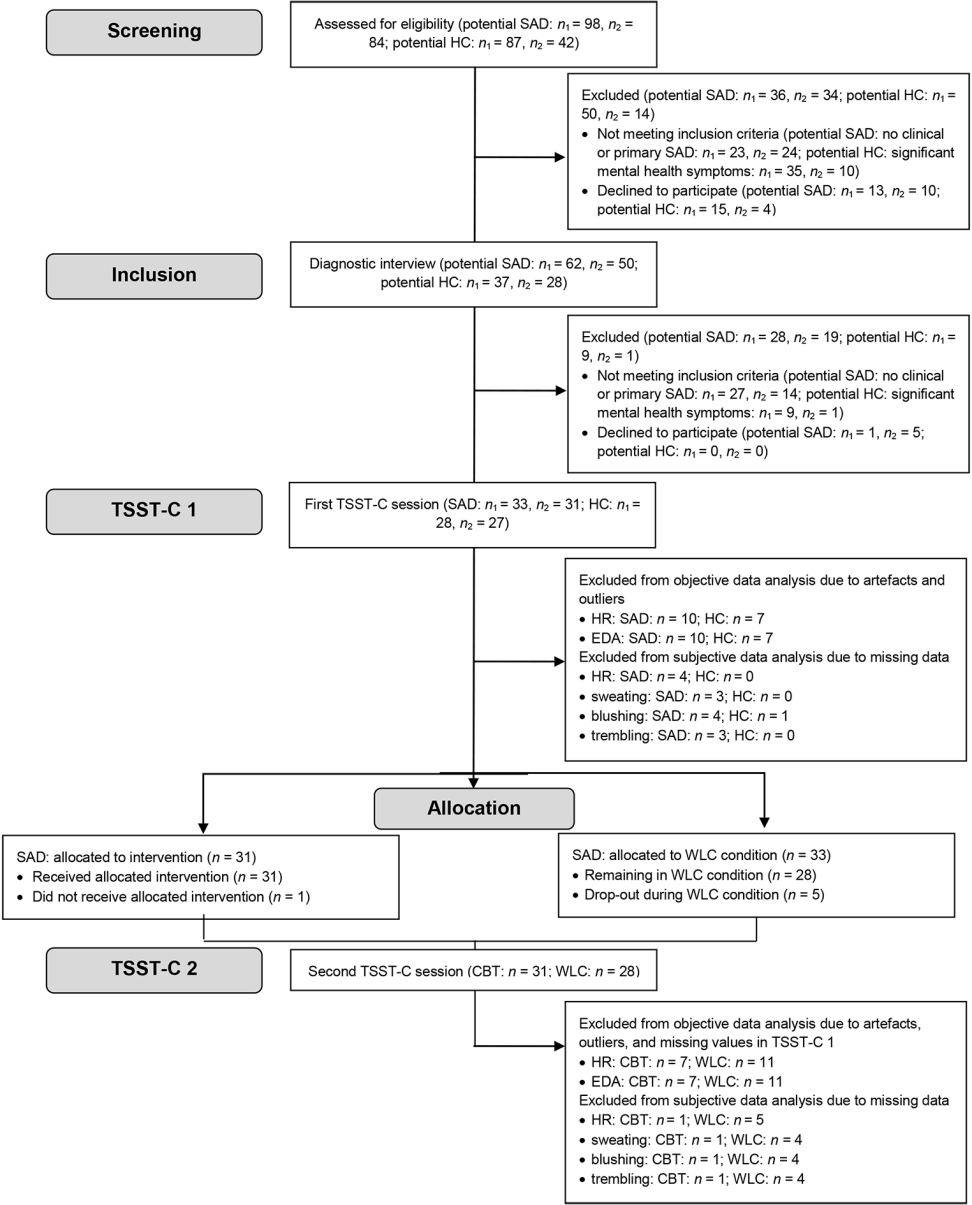
Flowchart of Study Participants *Note.* *n*_1_ = Center 1, *n*_2_ = Center 2; CBT = cognitive behavioral therapy; EDA = electrodermal activity; HC = healthy control; HR = heart rate; SAD = social anxiety disorder; TSST-C = Trier Social Stress Test for Children; WLC = waitlist control.

The study was designed as a randomized controlled trial (block randomization, in which half of the participants were allocated by drawing from a hat to an experimental condition receiving immediate treatment and half to a WLC condition receiving treatment about 16 weeks later; for an overview see Figure 1). Randomization for each research center was conducted in a concealed fashion by the other center, based on subject codes, as soon as there were enough participants for one experimental and one WLC allocation. Eligibility criteria were registered with the German Research Foundation (TU 78/5-2, HE 3342/4-2) prior to recruitment and not changed during the study. This study was part of a larger project. The overall project consisted of experimental studies related to research questions of visual attention allocation or psychophysiological processes under (social) stress and it also aimed to measure treatment success by including several outcome variables (state anxiety, negative cognitions, physiological arousal, perception of and worry about physiological symptoms, perception of academic performance, negative post-event processing, parental cognitions, parental fear of negative child evaluation, and related treatment outcome predictions). Due to the extent of the project and limitations on length and foci in articles, not all treatment related results could be reported in a single manuscript. Further results are reported elsewhere (treatment outcome, Asbrand, Heinrichs, Schmidtendorf, Nitschke, & Tuschen-Caffier, 2020; changes in post-event processing based on treatment, Asbrand, Schmitz, et al., 2019; stability of the cortisol response despite treatment, Asbrand, Heinrichs, Nitschke, Wolf, Schmidtendorf, & Tuschen-Caffier, 2019) or are being prepared for submission (social performance, detailed psychophysiological activity pre and post treatment). 

To ensure maximal transparency, all articles include cross-references to other reports on measures used to investigate potential treatment-related effects. The current study reports primary outcome variables relating to perception of (IS) and worry about physiological symptoms heart rate and EDA (IE). The inclusion of subjective perception of and worry about blushing and trembling as well as cardiac IAc was included post-hoc. The sample size for the current study, based on a medium to large effect (Schmitz et al., 2012) and power of (1 - β) = .80, was set at *n* = 90 (each group *n* = 45). As the study was part of a larger research project (see footnote) requiring a larger sample size of *n* = 110, all children were included to increase power. Because the data are being used in a large project, this Method section has been reported before in a similar fashion (Asbrand, Heinrichs, et al., 2019; Asbrand, Schmitz, et al., 2019).

### Participants

We informed parents of anxious children (9 to 13 years) through advertisements in schools, medical facilities, and newspapers in two midsized German cities from January 2012 to November 2013 until the targeted sample size had been reached (for an overview see Figure 1). No harms were reported. Parents received €35, and children €25 in vouchers in compensation for participation in the laboratory study. Ethical approval for this study was granted by an independent ethics committee (ethics committee of the German Society for Psychology). All participating children and their caregivers consented to participation in both oral and written form.

Inclusion criterion for children consisted of SAD as a primary diagnosis in the SAD group and no current or lifetime diagnosis of a mental disorder in the HC group. Exclusion criteria entailed health problems or medication which could have interfered with psychophysiological assessment (e.g., asthma, cardiac arrhythmia, and methylphenidate). As can be seen in Table 1, the groups did not differ in age, type of school, or any of the disorder-specific measures. Social Phobia and Anxiety Inventory for Children (SPAI-C) scores exceeded suggested cut-offs for clinically relevant SAD.

**Table 1:** Participant Characteristics of the Experimental Groups (Social Anxiety Disorder vs. Healthy Controls)

Characteristic	Group		Statistics
	SAD	Healthy controls	
*n* ^a^	64	55	
Mean age (*SD*), in years	11.3 (1.4)	11.3 (1.4)	*t*(117) = 0.06, n.s.
Female	63.6%	60.0%	χ^2^(1) = 0.17, n.s.
Mean SPAI-C (*SD*)	23.3 (9.03)	4.2 (5.4)	*t*(117) = -13.71***
Net income (per month)			χ^2^(8) = 11.42, n.s.
n.a.	0%	1.3%	
< €1,000	0%	5.9%	
€1,001–1,500	1.9%	7.4%	
€1,501–2,000	11.1%	8.8%	
€2,001–3,000	35.2%	32.4%	
€3,001–4,000	14.8%	16.2%	
€4,001–5,000	14.8%	20.6%	
> €5,000	22.2%	7.4%	
Mean (*SD*) state anxiety during TSST-C (before treatment)	6.6 (2.8)	4.5 (2.9)	*t*(117) = 4.05***

*Note.* Table adapted from Asbrand, Schmitz, et al. (2019). Reprinted with permission. SPAI-C = Social Phobia and Anxiety Inventory for Children; TSST-C = Trier Social Stress Test for Children; n.a. = not available.

^a^Sample sizes differ as not all questionnaires were completed correctly.

***p ≤ .001, n.s. = not significant.

Further, in the SAD group, children in the two conditions (CBT vs. WLC) did not differ in sociodemographic and psychopathological variables (see Table 2).

**Table 2:** Participant Characteristics of Children With Social Anxiety Disorder Allocated to the Treatment Versus Waitlist Group

Characteristic	Group		Statistics
	Treatment (CBT)	Waitlist control	
*n* ^a^	31	33	
Mean age (*SD*), in years	11.5 (1.4)	11.2 (1.3)	*t*(62) = 0.78, n.s.
Female	51.6%	67.6%	χ^2^(2) = 1.88, n.s.
Mean SPAI-C (*SD*)	11.8 (7.3)	12.1 (7.1)	*t*(62) = 0.18, n.s.
Net income (per month)			χ^2^(7) = 6.65, n.s.
n.a.	3.2%	0.0%	
< €1,000	6.5%	5.6%	
€1,001–1,500	9.7%	5.6%	
€1,501–2,000	6.5%	8.3%	
€2,001–3,000	41.9%	23.7%	
€3,001–4,000	16.1%	16.7%	
€4,001–5,000	9.7%	30.6%	
> €5,000	6.5%	8.3%	
Mean (*SD*) state anxiety during TSST-C (before treatment)	6.7 (2.9)	6.6 (2.8)	*t*(62) = 0.10, n.s.

*Note.* Table adapted from Asbrand, Schmitz, et al. (2019). Reprinted with permission. CBT = Cognitive behavioral therapy; n.a. = not available; SPAI-C = Social Phobia and Anxiety Inventory for Children; TSST-C = Trier Social Stress Test for Children.

^a^Sample sizes differ as not all questionnaires were completed correctly.

***p ≤ .001. n.s. = not significant.

### Procedure

The study took place at two German universities. All analyses first considered site differences, which were non-existent. Following a short telephone screening for anxiety symptoms, eligible children and their parents attended a diagnostic session (see flowchart in Figure 1). Both the child and a parent separately participated in the Kinder-DIPS, a structured interview that codes for mental disorders in children and adolescents (Schneider, Unnewehr, & Margraf, 2008). Diagnoses of SAD and comorbid disorders (*DSM-IV-TR*, APA, 2000) were then reached through combining both interviews, supervised by an experienced clinical psychologist. Diagnoses were assigned under supervision of the same licensed clinical psychologists per site throughout the project (one psychologist at the first, two psychologists at the second center). The Kinder-DIPS is a validated interview for the most frequent mental disorders in children and adolescents (Schneider et al., 2008). The Kinder-DIPS is conducted by trained interviewers and the diagnosis is usually based on both child and parent reports. The authors have reported adequate interrater reliability (87% for anxiety disorders), good retest reliability (Schneider et al., 2008), and successful validation with disorder-specific questionnaires. Additionally, children and parents reported sociodemographic data, anxiety symptoms, and general psychopathology in online questionnaires. According to the diagnostic assessment, 65 children fulfilled the inclusion criterion of a primary diagnosis of SAD; 55 children were included in the HC group.

**Figure 2:**
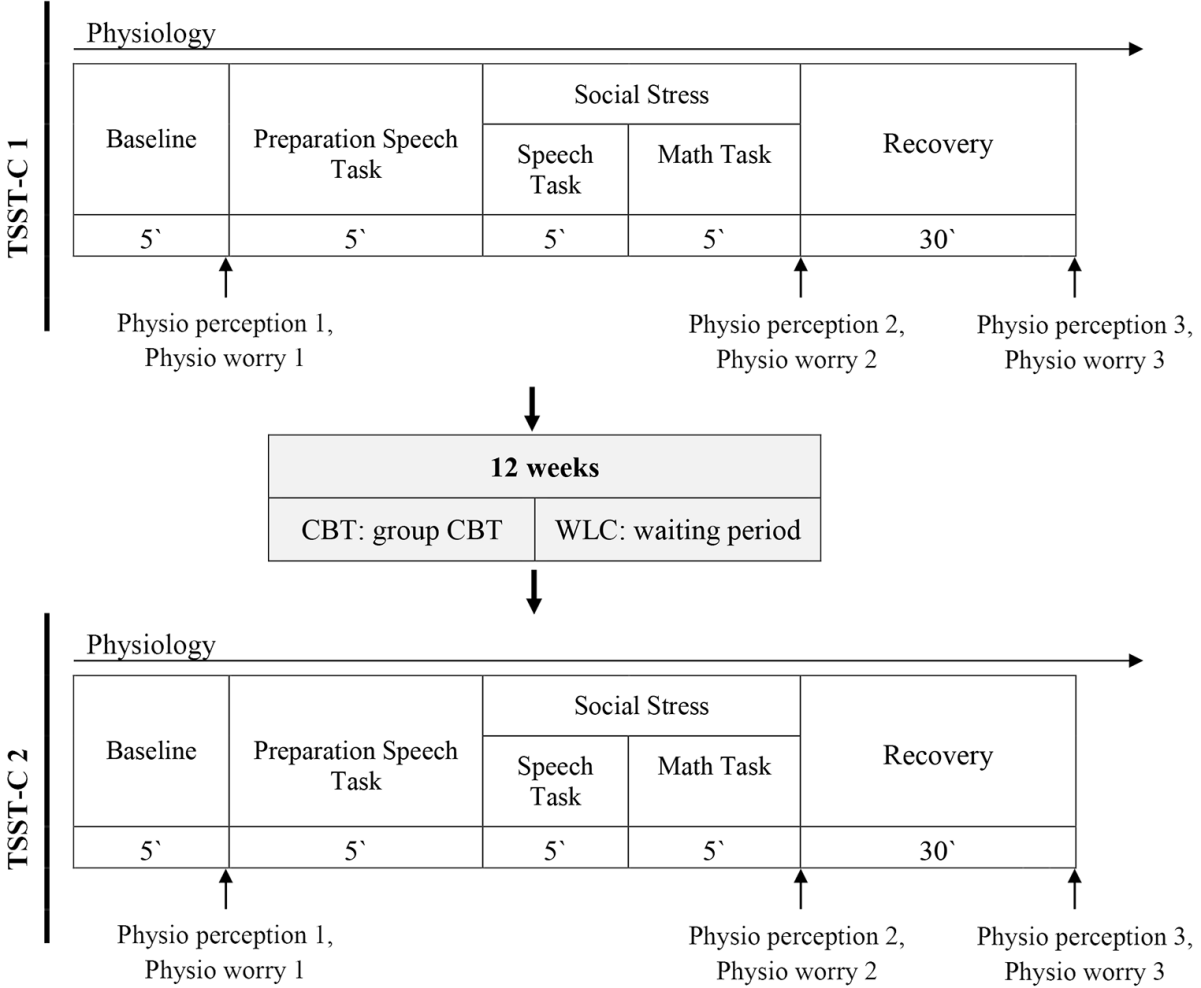
Overall Procedure Including the Trier Social Stress Test for Children (TSST-C) Before (TSST-C 1) and After (TSST-C 2) Treatment or Waiting *Note.* Physio perception 1–3 refers to measurements of participants’ perceived level of physiological intensity and physio worry 1–3 to worry about their physiological symptoms.

After the diagnostic interviews children participated in the first laboratory session, the TSST-C (Buske-Kirschbaum et al., 1997), which consists of a speech and a math task (see Figure 2). In the speech task, children narrate a story in front of two observers after listening to the beginning of the story. In the following mental arithmetic task, children were asked to serially subtract the number 7 from 758 (9- to 11-year-olds) or the number 13 from 1,023 (12- to 13-year-olds) as fast and as accurately as possible again in front of two observers. Both observers were instructed and trained to give neutral verbal and nonverbal feedback. The TSST-C elicits high social-evaluative stress in children (cf. Allen et al., 2017). Throughout the session, heart rate and skin conductance level were assessed. Further, perception of (IS) and worry about physiological symptoms (IE) were assessed after baseline, stress, and recovery (see Figure 2). After a recovery period, children performed the HCT to assess IAc (see below). As the current project focused on the climax of social stress, only this time of measurement was included in the analyses. Assessments of perception and worry were based on a previous study (Schmitz et al., 2012): Children were asked to rate their perceived level of physiological intensity during the task (e.g., “How strongly did you feel your heartbeat during the task?”) and their worry about their physiological symptoms (“How much did you worry that others could notice how fast your heart was beating?” on a scale of 0 (*not at all*) to 10 (*extremely*). After participating in a 12-week CBT program (CBT group) or waiting without treatment (WLC group), all children performed a parallel version of the first testing session. Based on the original TSST-C (Buske-Kirschbaum et al., 1997), the speech task was changed to a different story that was judged to be similarly interesting and difficult in a preevaluation. The math task was changed to a different start number (+10). The TSST-C reliably induces social anxiety in all children, even more so in children with SAD compared to healthy control children, *p* < .001.

### Treatment

Treatment consisted of an exposure-based CBT treatment that was evaluated simultaneously (Asbrand, Heinrichs, Schmidtendorf, Nitschke, & Tuschen-Caffier, 2020). It targets dysfunctional cognitions, possible social deficits, and social avoidance with a strong focus on exposure. Each session consisted of 100 min (including a 10-min break) in groups of five to seven children. Standard CBT components were implemented in 12 sessions (psychoeducation, cognitive restructuring, social skills training, exposure, and relapse prevention). Children were instructed to use their newly developed skills outside of treatment to ensure a transfer into everyday life.

### Psychometric Measure

The SPAI-C (Beidel, Turner, Hamlin, & Morris, 2000) assesses behavioral characteristics specific to SAD (26 items; e.g., “I am anxious when I meet new boys or girls”). Children respond to each item using a 3-point Likert-type scale ranging from “never or hardly ever” to “almost always or always.” Validity and reliability were confirmed in the original sample (Beidel et al., 2000) and a German sample (Melfsen, Walitza, & Warnke, 2011). Internal consistency and test–retest reliability after 4 weeks in the German sample was excellent (Cronbach’s α = .92; *r*_tt_ = .84).

### Psychophysiological Measures

Electrodermal and cardiovascular measures including heart rate were assessed at 400 Hz using the Varioport system (Becker Meditec, Karlsruhe, Germany). Data inspection and artefact rejection were conducted offline using ANSLAB (Blechert, Peyk, Liedlgruber, & Wilhelm, 2016). For the electrocardiogram, the cardiac interbeat interval (IBI), calculated as the interval in milliseconds between successive R waves, was extracted. For illustrative purposes the IBI was converted to heart rate (in beats per minute) for tables and figures but all statistical analyses were based on IBI values (Quigley & Berntson, 1996). EDA, reflecting electrodermal sympathetic activity (Boucsein, 2012), was assessed by placing two electrodes on the middle phalanx of the middle and ring fingers of the left hand using 11-mm inner diameter Ag/AgCl electrodes filled with isotonic electrode paste (TD-245, Med Associates, Inc., St. Albans, Vermont). As a parameter of EDA, skin conductance level was used.

### Interoceptive Accuracy (IAc)

We assessed IAc using the HCT. After a short training of about 10s, children were asked to silently count their heartbeats during three instructed intervals (25, 35, 45s in a fixed order), to indicate ‘zero’ if they had not perceived any, and not take their pulse or to use any other strategies such as holding their breath (Eley et al., 2004). Subjective reports of perceived heartbeats were checked for plausibility. For the first testing session, perceived heart beats ranged between 10 and 86 (25s interval), 10 and 90 (35s interval), and 13 and 600 (45s interval). Based on the extreme value at the third interval, one child was excluded from further analyses as it is possible that the child did not understand the instructions correctly, leaving a range between 13 and 120 (45s interval). For the second testing session, perceived heart beats ranged between 7 and 70 (25s interval), 1 and 85 (35s interval), and 6 and 105 (45s interval).

To ensure comparability to an earlier study (Koch & Pollatos, 2014), IAc was calculated using the formula:


$${\text{IAc}}_{\mathrm{HCT}}=\;\frac{1}{3}\;\sum_{k=1}^{3}\left(1-\frac{\left|{\text{no. of recorded heartbeats}}_{k}-{\text{no. of perceived heatbeats}}_{k}\right|}{{\text{no. of recorded heartbeats}}_{k}}\right)$$

Higher scores indicate higher IAc, with a maximum score of ‘1’ reflecting perfect IAc. As physical symptom reporting is related to the tendency to report false alarms in a somatosensory signal detection task (Brown et al., 2012), we calculated a simple IAc score to distinguish over- from underreporting using the formula (Rost, Van Ryckeghem, Schulz, Crombez, & Vögele, 2017):


$${\text{IAc}}_{\text{simple}}=\;\frac{1}{3}\;\sum_{k=1}^{3}\left(\frac{{\text{no. of perceived heartbeats}}_{k}-{\text{no. of recorded heartbeats}}_{k}}{{\text{no. of recorded heartbeats}}_{k}}\right)$$

A positive score reflects over-reporting and a negative score reflects underreporting of heartbeats.

### Statistical Analysis

First, for objective measures, statistical outliers 2.5 SD above or below the mean were excluded. Outliers were calculated separately for groups and time. To examine biases before treatment, we conducted analyses of variance (ANOVAs) with repeated measures on phase (baseline, stress, recovery), using group (SAD, HC) as between-subjects factor. For objective physiology, EDA and heart rate were used as dependent variables in separate ANOVAs. For subjective perception, rating (perception, worry) was further added as a factor. We included first the heart rate and perspiration scales and then the blushing and trembling scales as dependent variables in separate ANOVAs. Including objective physiology as a covariate did not lead to any significances, *p*s > .05, and is therefore not further reported. To consider that objective physiology and subjective perception (IS) and worry (IE) might depend on each other, multiple correlation analyses were conducted for both EDA and heart rate including subjective and objective measures. IAc scores (IAc_HCT_ and IAc_simple_) from the first laboratory session were compared using an independent sample *t* test with *group* (SAD, HC) as independent variable.

For treatment effects, we once again conducted ANOVAs with repeated measures on session (pre, post) and phase (baseline, stress, recovery), using group (CBT, WLC) as between-subjects factor. For objective physiology, EDA and heart rate were used as dependent variables in separate ANOVAs. For subjective perception, perception and worry of all physiology questionnaires (heart rate, perspiration, blushing, trembling) were analyzed as dependent variables in separate ANOVAs. Once again, multiple correlation analyses were conducted for both EDA and heart rate including subjective and objective measures. For the analysis of treatment effects on IAc scores, an ANOVA with repeated measures on *time* (pre, post) was used based on *treatment* (CBT, WLC) as independent variable. Further, a moderation analysis was conducted testing treatment as potential moderator (CBT, WLC) between IAc pre (IAc_1) and post treatment (IAc_2) using the PROCESS macro for SPSS (Hayes, 2013). Further exploratory analyses are reported in the Supplementary Materials.

Significant main effects and interactions for all ANOVAs were further analyzed with post hoc *t* tests for independent groups for the group comparisons and with *t* tests for dependent groups for the time comparisons (phase, session) if relevant for the hypotheses. Cohen’s *d* effect sizes are reported for the post hoc tests.

## Results

### Before Treatment: Objective Physiology Comparison of Children With and Without SAD

**Figure 3:**
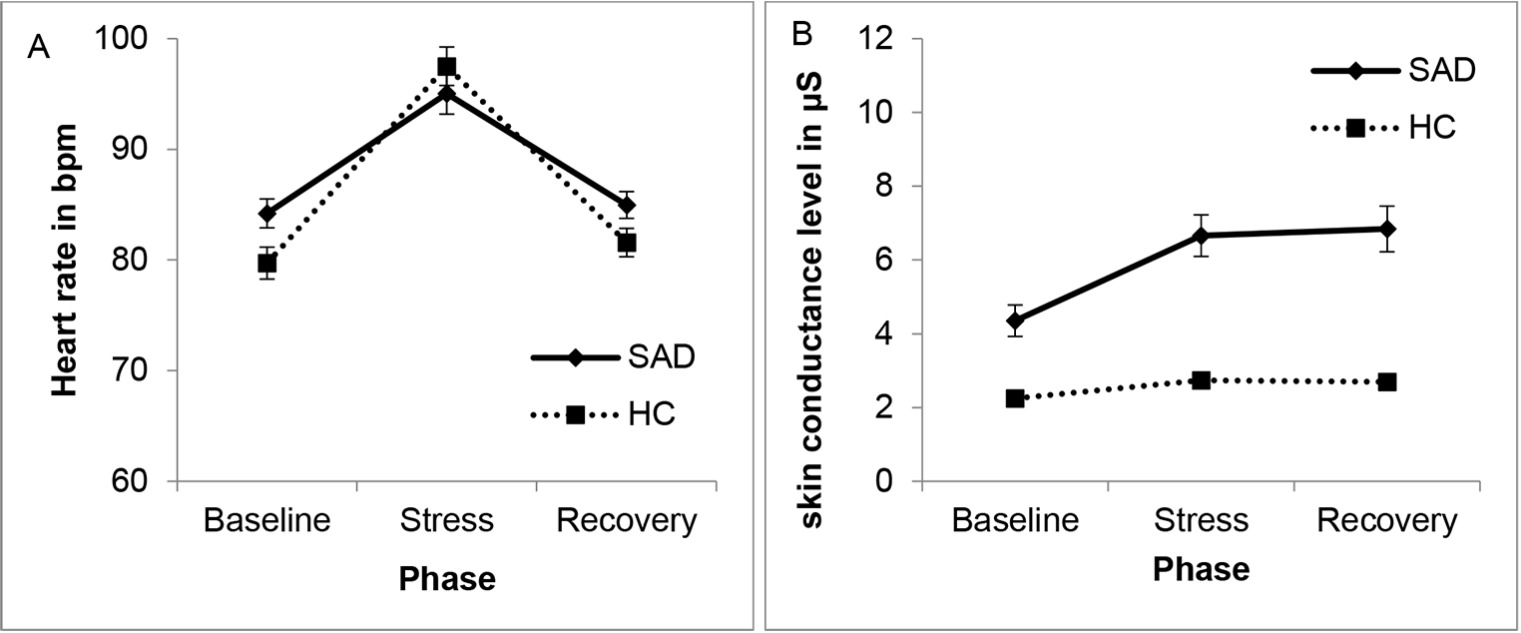
Group Comparisons of (A) Heart Rate (in Beats per Minute, BPM) and (B) Electrodermal Activity During the First Trier Social Stress Test for Children for Children With Social Anxiety Disorder (SAD) and Healthy Control (HC) Children

We found higher heart rate (HR) during the stress as compared to the baseline and post phases, Wilk’s λ = .351, *F*(2,94) = 87.07, *p* < .001, $\eta_{p}^{2}$ = .649, but HR did not differ between groups, *F*(1,95) = 0.87, *p* = .354. There was a significant interaction of Phase × Group, Wilk’s λ = .870, *F*(2,94) = 87.07, *p* < .001, $\eta_{p}^{2}$ = .130. Post hoc tests showed a significantly higher HR in children in the SAD group during the baseline phase, *t*(95) = -2.30, *p* = .023, *d* = 0.47, but no further group differences, *t*s < 1.33, *p*s > .187 (see Figure 3A). In the SAD group, HR increased significantly from baseline to stress, *t*(53) = 7.12, *p* < .001, *d* = 0.41, and decreased from stress to recovery, *t*(53) = -7.27, *p* < .001, *d* = 0.40. Similarly, in the HC group, HR increased significantly from baseline to stress, *t*(42) = 11.31, *p* < .001, *d* = 0.67, and decreased from stress to recovery, *t*(53) = -10.40, *p* < .001, *d* = 0.61. The significant interaction between group and phase and the higher effect sizes for post-hoc tests in the HC than the SAD group suggest a steeper increase and decrease in the HC group compared to the SAD group.

EDA significantly increased over time, Wilk’s λ = .586, *F*(2,91) = 32.17, *p* < .001, $\eta_{p}^{2}$ = .414, and differed between groups, *F*(1,92) = 35.12, *p* < .001, $\eta_{p}^{2}$ = .276. Furthermore, we observed a significant Phase × Group interaction, Wilk’s λ = .750, *F*(2,91) = 15.14, *p* < .001, $\eta_{p}^{2}$ = .250. Post hoc tests showed that in the SAD group, EDA increased significantly from baseline to stress, *t*(49) = 7.17, *p* < .001, *d* = 0.31, but did not decrease from stress to recovery, *t*(49) = 1.30, *p* = .199, *d* = 0.02. Similarly, in the HC group, EDA increased significantly from baseline to stress, *t*(43) = 4.45, *p* < .001, *d* = 0.23, but did not decrease from stress to recovery, *t*(43) = 1.02, *p* = .311, *d* = 0.02 (see Figure 3B). Again, the significant interaction and the higher effect sizes imply a steeper increase in the HC group compared to the SAD group.

### Before Treatment: Subjective Physiology Perception Comparison of Children With and Without SAD

**Figure 4:**
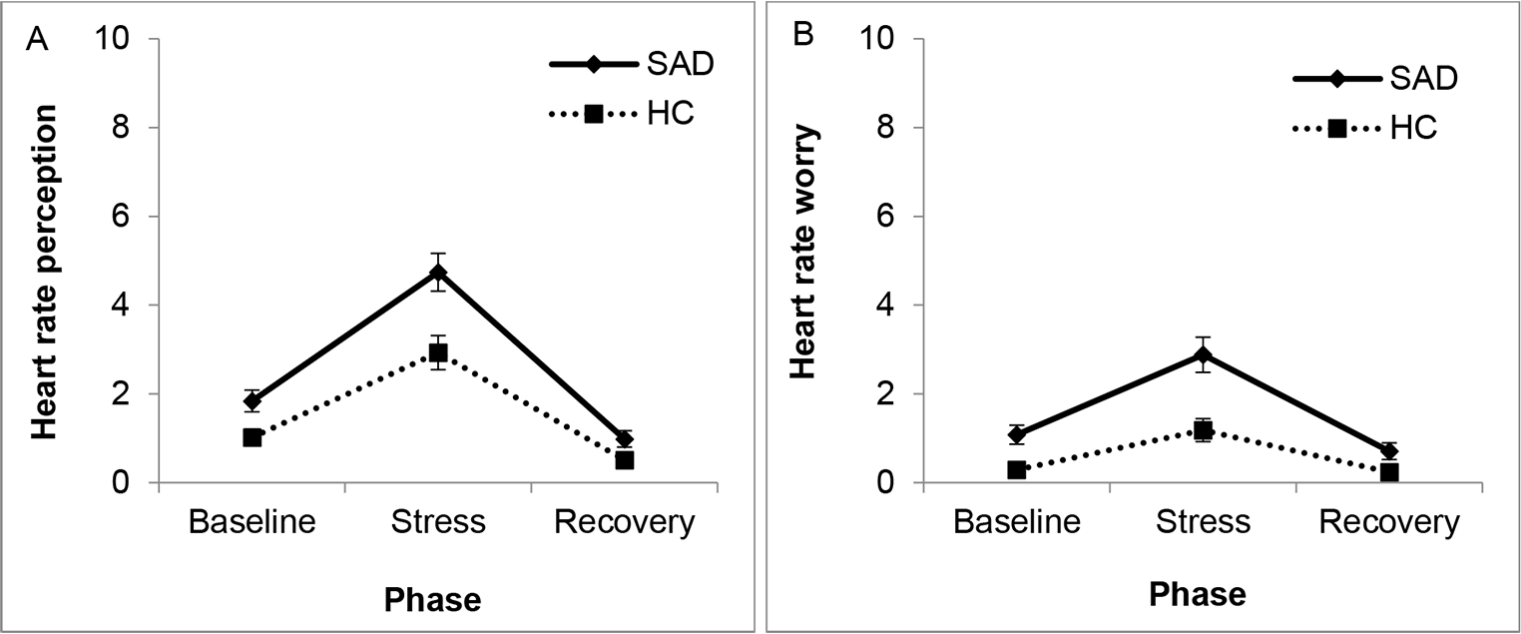
Subjective Perception of (A) and Worry (B) About Heart Rate After All Phases of the First Trier Social Stress Test for Children *Note.* For other parameters, see Supplementary Materials.

For subjective perception of heart rate (IS), we found significant main effects of phase, Wilk’s λ = .468, *F*(4,111) = 31.49, *p* < .001, $\eta_{p}^{2}$ = .532, and group, Wilk’s λ = .861, *F*(2,113) = 9.10, *p* < .001, $\eta_{p}^{2}$ = .139, with a trend for a significant interaction of Phase × Group, Wilk’s λ = .929, *F*(4,111) = 2.11, *p* = .084, $\eta_{p}^{2}$ = .071. Groups differed in both perception of and worry about heart rate in all phases (see Figure 4; *p*s < .05). The increase from baseline to stress and the decrease from stress to recovery was significant in both groups, *p*s < .001. Similar effects were found for subjective perception of perspiration, blushing, and trembling (see Supplementary Materials).

### Before Treatment: Relations Between Objective and Subjective Physiology

A multiple correlation analysis between objective heartrate and subjective perception of and worry about heart rate did not reveal any significant correlation, *p*s > .084. Similarly, no effects were found for EDA and subjective perception, *p*s > .105.

For the first laboratory session, neither the IAc_HCT_ scores, *t*(96) = -1.29, *p* = .200, *d* = 0.26, nor the IAc_simple_ scores differed significantly between groups, *t*(98) = -1.48, *p* = .142, *d* = 0.30.

### After Treatment: Objective Physiology Comparison of Children With SAD After Treatment Versus Waiting

Comparable to the first measurement occasion, HR was higher during stress than during baseline and post phases, Wilk’s λ = .355, *F*(2,37) = 33.55, *p* < .001, $\eta_{p}^{2}$ = .645. All other effects remained nonsignificant, *F*s < 2.77, *p*s < .103.

Again, EDA was higher during stress than during baseline and post phases, Wilk’s λ = .388, *F*(2,37) = 29.15, *p* < .001, $\eta_{p}^{2}$ = .612. All other effects remained nonsignificant, *F*s < 3.91, *p*s < .057.

### After Treatment: Subjective Physiology Perception Comparison of Children With SAD After Treatment Versus Waiting

**Figure 5:**
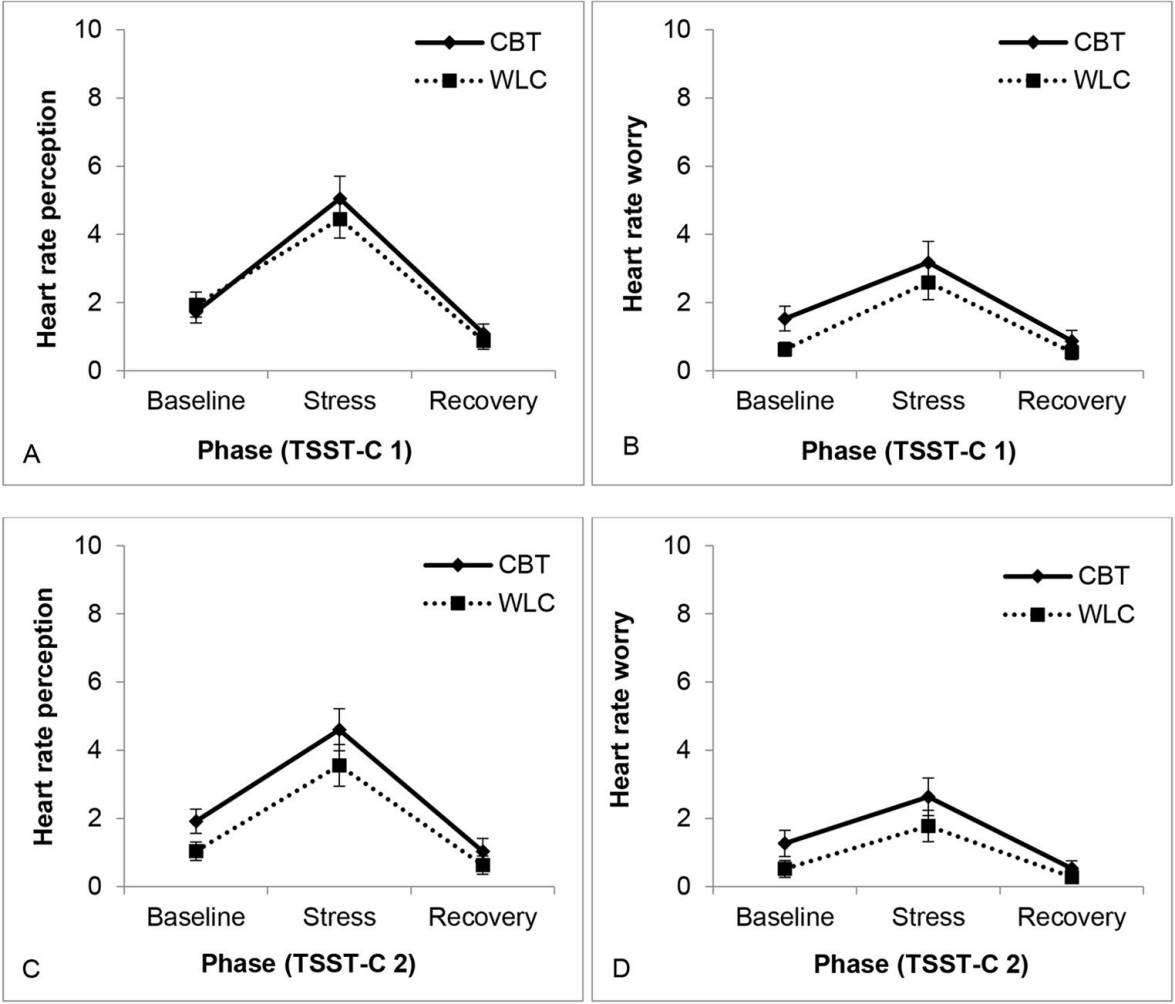
Subjective Perception of and Worry About Heart Rate After All Phases of the First (A, B) and Second (C, D) Trier Social Stress Test for Children (TSST-C), Comparing the Cognitive Behavioral Therapy (CBT) and Waitlist Control (WLC) Groups *Note.* For other parameters, see Supplementary Materials.

For subjective perception of heart rate after treatment, the ANOVA showed a significant main effect of phase, Wilk’s λ = .364, *F*(4,48) = 20.94, *p* < .001, $\eta_{p}^{2}$ = .636, and a trend for a significant effect of session, Wilk’s λ = .891, *F*(2,50) = 3.07, *p* = .055, $\eta_{p}^{2}$ = .109. All other *F*s < 2.27, *p*s > .113 (see Figure 5).

An analysis of the main effect of session for heart rate in both groups, using *t* tests for paired samples, showed an overall decrease in the perception, *t*(56) = 2.03, *p* = .047, *d* = 0.28, and worry after the stress phase, *t*(56) = 2.22, *p* = .030, *d* = 0.30. All other *t*s < 1.33, *p*s > .191. So, heart rate perception and worry decreased in all children from TSST-C 1 to TSST-C 2. Similar effects were found for subjective perception of trembling (see Supplementary Materials).

### After Treatment: Relations Between Objective and Subjective Physiology

A multiple correlation analysis at TSST-C 2 between objective HR and subjective perception (IS) of and worry (IE) of HR did not reveal any significant correlation, *p*s > .077. Similarly, no effects were found for EDA and subjective perception, *p*s > .229.

Regular IAc_HCT_ did not change from pre- to post-measurement (main effect ‘session), independent of treatment group (interaction treatment × session, *F*s < 1.92, *p*s > .174). Likewise, for IAc_simple_, no significant effects appeared for treatment, session or session × treatment, *F*s < 2.19, *p*s > .146.

Additionally, the moderation analysis showed an overall significant relation, *R*^2^ = .382, *F*(3,35) = 7.2, *p* < .001. There was a significant relation between IAc_1 and IAc_2, while treatment was no significant moderator (Table 3).

**Table 3:** Prediction of IAc at Second Laboratory Session

Predictor	*b*	*SE B*	*t*	*p*
Constant	-0.12 [-0.75, 0.51]	0.31	-0.38	.703
IAc_1 (standardized)	0.92 [0.03, 1.18]	0.44	2.09	.044
Treatment (CBT, WLC; standardized)	0.27 [-0.19, 0.74]	0.23	1.20	.237
IAc_1 × Treatment (CBT, WLC)	-0.25 [-0.90, 0.40]	0.32	-0.79	.437

*Note*. IAc_1 = Interoceptive accuracy laboratory session 1, IAc_2 = Interoceptive accurarcy laboratory session 2.

## Discussion

The study aimed to assess alterations in perception of (IS) and worry about (IE) physiological symptoms as well as IAc in childhood SAD. It further strived to examine possible changes after CBT. Supporting our hypotheses at TSST-C 1, children with SAD showed higher heart rate than children in the HC group during the baseline phase, and a lower reactivity to stress. Further, EDA was heightened throughout the testing session. These findings may indicate an autonomic hyperarousal and blunted stress reactivity in the SAD group. Moreover, children in the SAD group reported heightened perception (IS) of and increased worry (IE) about heart rate, perspiration, and trembling throughout the TSST-C. There seems to be no biased perception for EDA. However, the pattern for the objective and subjective side in heart rate differed: Objectively, children in the HC group showed a steep increase and decrease throughout the TSST-C 1. Subjectively however, HC children’s perception and worry remained below that of SAD children. As blushing and trembling were not controlled on objective parameters, this effect can only be confirmed for heart rate and perspiration. Further, contrary to findings in adults (Domschke, Stevens, Pfleiderer, & Gerlach, 2010), no differences in IAc were found between children with SAD and HC children.

Findings after treatment were not in line with our hypotheses: Objective physiological parameters (heart rate, EDA) did not change. Interestingly, children in the CBT group reported heightened perception of and increased worry about perspiration, and trembling after the baseline phase at TSST-C 2 compared to children in the WLC group. Additionally, both before and after treatment subjective and objective parameters did not correlate. Further, as no differences appeared between children with SAD receiving treatment vs. waiting, no effects of treatment on IAc can be assumed.

### Before Treatment: Findings on Children With SAD Compared to an HC Group

Objectively in line with earlier studies (cf., Asbrand et al., 2017), a tonic hyperarousal was shown in children with SAD concerning EDA. However, in contrast to earlier studies (Schmitz et al., 2012), this was mirrored by subjective perception. Part of the earlier findings in high socially anxious children (Schmitz et al., 2012) still seems to be also found in our sample: Concerning HR, children with SAD perceived an increase in their physiological reaction that was not mirrored by the pattern of physiological reactivity. Further, they worried more than children in the HC group that this physiological arousal might be observable. Considering the point of (non)visibility of heart rate, children with SAD might have more unrealistic worries that internal signals might be observable. As our paradigm was slightly altered to Schmitz et al. (TSST-C instead of a speech task with public vs. private sound of heart rate), our findings are not replication in a narrow sense but show a robust effect in an established social stress test. However, mean scores on symptom perception intensity as well as worry were rather low (< 5 on a scale of 0 to 10). The pattern of results demonstrates that worry is linked to several physiological symptoms (but not all). Further, it may be that some physiological sensations are more likely linked to SAD (e.g., blushing; Bögels, Rijsemus, & De Jong, 2002). These symptoms are more consistently associated with worry, reflecting a more general tendency to worry about SAD-related physiological symptoms instead of symptom-specific links between perception of and worry about these symptoms. This might be related to visibility of physiological symptoms. Finally, a lack of correlation between IAc, IS and IE provides an interesting insight: It would be expected that a higher physiological arousal leads to the perception of – and possibly worry about – these symptoms. However, our results point to the independence of both sides. This might stem from the fact that children struggle more with IAc (Koch & Pollatos, 2014). The current study suggests that SAD children do not show altered IAc, but report higher subjective heart rates (IS) and higher worries about cardiac perceptions (IE). SAD in childhood may be reflected, therefore, by a selective increase in the subjective tendency to be focused on heart rate increases and a negative evaluation of these percepts, whereas the actual perception is unaffected.

### After Treatment: Findings on a Treatment (CBT) Versus a WLC Group

Children in the CBT group reported higher perception of and more worry about perspiration as well as more worry about trembling after the baseline phase of TSST-C 2. In other words, children in the CBT group reported heightened perception of physiological arousal and increased worry on some parameters after the baseline phase. Previous findings from this sample could show that CBT was in general successful in reducing the severity of SAD as measured by a blind interview after treatment (cf., Asbrand et al., 2020). Further, some SAD-relevant rumination processes such as post-event processing changed for the better as negative thoughts after a social situation decreased significantly after treatment (Asbrand, Schmitz, et al., 2019). Still, cortisol levels did not change based on treatment; however, cortisol levels in the WLC group increased in the second TSST-C (Asbrand, Heinrichs, et al., 2019). Overall, it would have been plausible that physiological awareness and biased perception also change with treatment. However, instead of decreasing, children in the CBT group reported higher perception and worry about several physiological parameters before entering the social stress situation. It might be that children in the CBT group were sensitized to similar tasks as they had experienced exposure sessions beforehand. Psychoeducation conveys a concept of anxiety that includes cognitions, behavior, and physiological reactions. Often, this is the first time children are confronted with such a concept. It might direct their attention to these factors and, as such, support sensitization. Further, our treatment was rather short (12 sessions), and recent research has argued that longer treatment is necessary in SAD (e.g., Hudson et al., 2015). As the main treatment component, exposure, had to be properly prepared (habituation rationale, first exposure in social skills sessions), only a few sessions remained to experience in-vivo exposure. Thus, it is possible that treatment was already successful in reducing overall symptoms (Asbrand, Heinrichs, et al., 2020), but children were still in the process of handling high state anxiety. Additionally, our treatment did not specifically target physiological symptoms and their interpretation. This treatment component is more common in treatment of panic disorders (e.g., Clark et al., 1999; Öst & Westling, 1995) but should be considered for SAD treatment as well, given our results. However, interpretation of these findings of elevated perception and worry in the CBT group should be evaluated cautiously as they were found only after the baseline phase of the TSST-C and not after the stress phase. In addition, even if the pattern of results allows for interpretation of sensitization, the overall scores remain low at posttreatment (mean scores < 4 on a scale of 0 to 10).

Finally, while comparison of single intervals of the HCT has shown high correlations between these (e.g., Koch & Pollatos, 2014), our study is the first to show stability over a longer period of time providing first evidence for IAc as a trait marker in children. However, as we do not find differences in IAc between children with and without SAD in our study, IAc may not play a key role for SAD in children. Possibly, a subsample of children with SAD suffering from panic-like symptoms (cf. Domschke, Stevens, Pfleiderer, & Gerlach, 2010) in social situations might show both different IAc scores and changes in IAc based on treatment. Future studies are warranted to investigate, which role other occasion- or situation-specific factors, as well as error variance (Wittkamp et al., 2018), contribute to IAc in in children.

### Limitations and Conclusions

While the study has several strengths, such as a clinical sample and inclusion of treatment, several limitations apply. First, we assessed a variety of dependent variables based on concerns to target physiological arousal broadly (Siess et al., 2014). Possibly, a lack of effects might stem from lack of power. However, the current sample was relatively large and could detect differences in treatment groups, even though they showed to be contrary to expectations. Second, we did not assess all variables both subjectively and objectively but provide subjective data only for blushing and trembling. Future studies might target these variables to examine the objective basis for subjective perception. Previous studies from adults, however, point to similar results for blushing, as this depends mainly on social anxiety instead of objective blood flow (Drummond & Su, 2012). Third, we refrained from using an experimental setup (cf., Gerlach et al., 2004; Schmitz et al., 2012), instead opting for a standardized social stress task. Thus, taking note of these earlier findings (Gerlach et al., 2004; Schmitz et al., 2012) on the importance of the perception of and worry about physiological arousal in social anxiety, we did not manipulate visibility of physiological arousal but chose to measure subjective and objective arousal in parallel during social stress. Finally, we did not include a correlation analysis between a change in social anxiety symptoms and changes in perceptions of physiology as this would not have been based on a solid theoretical background. However, future studies could include this perspective to analyze a possible co-occurrence of change in anxiety and perception of physiology.

In conclusion, our results indicate that SAD children show a selective enhancement of subjective cardiac interoception, as proposed by cognitive models of SAD (Clark & Wells, 1995), whereas behavioral indices of cardiac interoception and the perception of EDA changes remain unaffected. CBT did not change this perception. Thus, further inclusion of treatment components targeting this bias as currently proposed mainly by adult research (Hofmann & Otto, 2017; Naim, Kivity, Bar-Haim, & Huppert, 2018; Wong et al., 2017) should be considered.

## Supplementary Materials

The supplementary materials include additional exploratory analyses on subjective perception of perspiration, blushing and trembling before and after treatment (for access see Index of Supplementary Materials below):

10.23668/psycharchives.3086 Supplement 1 Supplementary materials to "Biased perception of physiological arousal in child social anxiety disorder before and after cognitive behavioral treatment"

AsbrandJ.
SchulzA.
HeinrichsN.
Tuschen-CaffierB.
 (2020). Supplementary materials to "Biased perception of physiological arousal in child social anxiety disorder before and after cognitive behavioral treatment"
[Additional exploratory analyses]. PsychOpen. 10.23668/psycharchives.3086PMC964549236397826
