# Supplementary material for: Biased Perception of Physiological Arousal in Child Social Anxiety Disorder Before and After Cognitive Behavioral Treatment
Source: Clin Psychol Eur. 2020 Jun 30;2(2):e2691. doi: 10.32872/cpe.v2i2.2691 (PMC9645492; doi:10.32872/cpe.v2i2.2691)
Supplement: Supplement 1 [file cpe-02-2691-s1.pdf]

Before treatment: Subjective physiology perception comparison of children with and without SAD

For subjective perception of perspiration, the analysis of variance (ANOVA) showed significant main effects of phase, Wilk's $\lambda = .607$, $F_{(4,112)} = 18.12$, $p < .001$, $\eta_p^2 = .393$, and group, Wilk's $\lambda = .830$, $F_{(2,114)} = 11.68$, $p < .001$, $\eta_p^2 = .170$. Further, a trend for a significant interaction effect were found for Phase × Group, Wilk's $\lambda = .926$, $F_{(4,112)} = 2.23$, $p = .071$, $\eta_p^2 = .074$. Similar to heart rate results, groups differed in both perception of and worry about perspiration after all phases (see Figure S1, *p*s < .05). The increase from baseline to stress and the decrease from stress to recovery was significant in both groups, *p*s < .001.

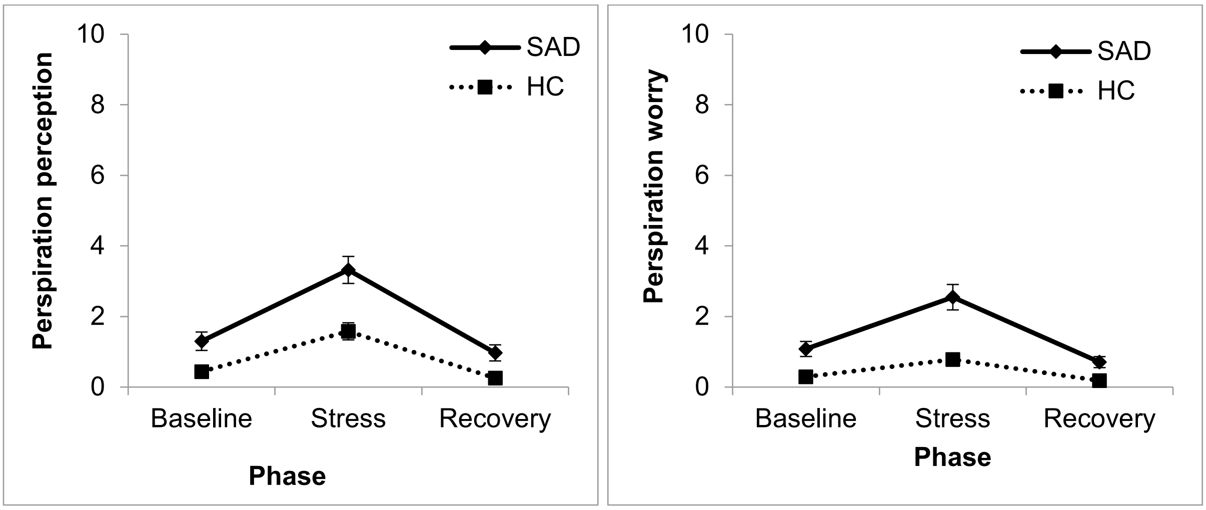

Figure S1. Subjective perception of and worry about perspiration after all phases of the first Trier Social Stress Test for Children. SAD = Group of children with social anxiety disorder; HC = healthy control children.

For subjective perception of blushing, the ANOVA showed significant main effects of phase, Wilk's $\lambda = .613$, $F_{(4,110)} = 17.34$, $p < .001$, $\eta_p^2 = .387$, and group, Wilk's $\lambda = .945$, $F_{(2,112)} = 3.25$, $p = .042$, $\eta_p^2 = .055$. The interaction Phase × Group did not reach significance, $p = .163$. For worry about blushing, post hoc *t* tests showed a significant difference between groups, with higher worry about blushing after the stress phase in the SAD group compared to the HC group, $t_{(104.9)} = 2.67$, $p = .009$, $d = 0.49$ (see Figure S2). All other *t*s < 1.64, *p*s < .105.

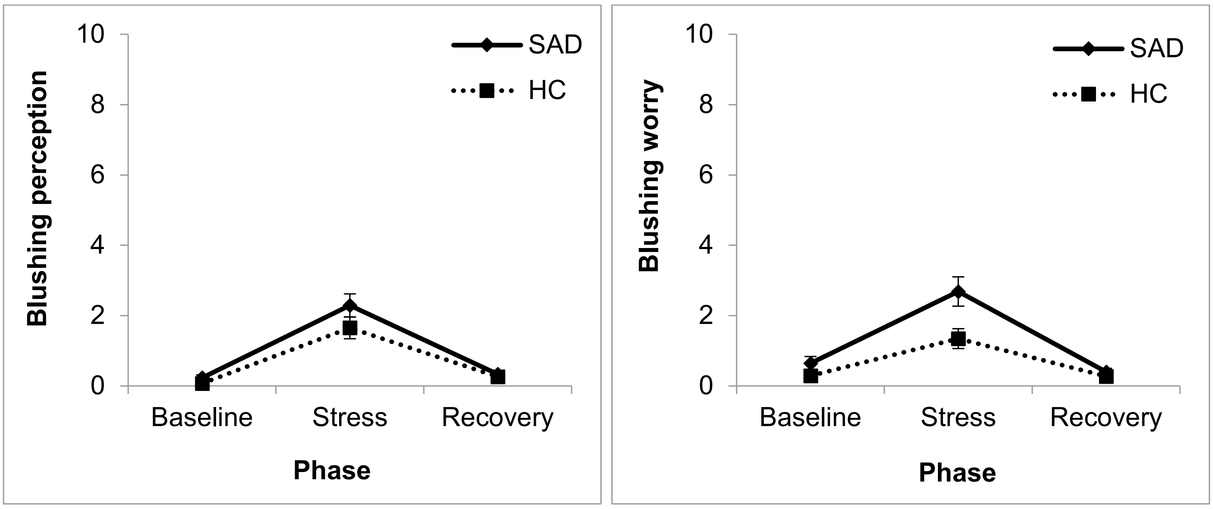

Figure S2. Subjective perception of and worry about blushing after all phases of the first Trier Social Stress Test for Children. SAD = Group of children with social anxiety disorder; HC = healthy control children.

For subjective perception of trembling, the ANOVA showed significant main effects of phase, Wilk's λ = .581, $F_{(4,112)} = 20.17$, $p < .001$, $\eta_p^2 = .419$, and group, Wilk's λ = .817, $F_{(2,114)} = 12.75$, $p < .001$, $\eta_p^2 = .183$. Further, a significant interaction was found for Phase × Group, Wilk's λ = .915, $F_{(4,112)} = 2.59$, $p = .041$, $\eta_p^2 = .085$. Post hoc *t* tests showed a significant difference between groups for both perception of and worry about trembling after all phases (see Figure S3, *p*s < .05). The increase from baseline to stress and the decrease from stress to recovery was significant in both groups, *p*s < .001. In sum, while there are group differences, the reported perception, as well as the related worry, is rather small to moderate overall across both groups.

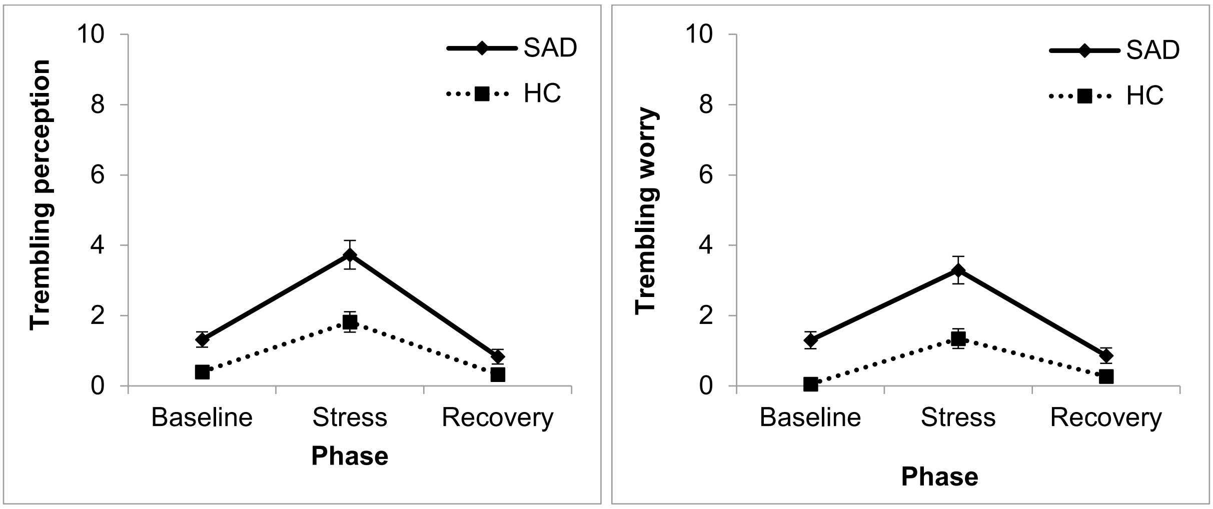

Figure S3. Subjective perception of and worry about trembling after all phases of the first Trier Social Stress Test for Children. SAD = Group of children with social anxiety disorder; HC = healthy control children.

After treatment: Subjective physiology perception comparison of children with SAD after treatment versus waiting

For subjective perception of perspiration after treatment, the ANOVA showed significant main effects of phase, Wilk's $\lambda = .522$, $F_{(4,49)} = 11.21$, $p < .001$, $\eta_p^2 = .478$, and group, Wilk's $\lambda = .889$, $F_{(2,51)} = 3.18$, $p = .050$, $\eta_p^2 = .111$. All other Fs < 1.16, ps > .331. Post hoc t tests for independent samples showed a significantly higher perception of, $t_{(37.3)} = 3.10$, $p = .004$, $d = 0.81$, and worry about, $t_{(37.3)} = 2.99$, $p = .005$, $d = 0.78$, perspiration at TSST-C 2 in the CBT group after the baseline phase (see Figure S4). All other ts < 1.86, ps > .069. After the baseline phase posttreatment, children in the CBT group reported higher perception of and worry about the visibility of perspiration than children in the WLC group.

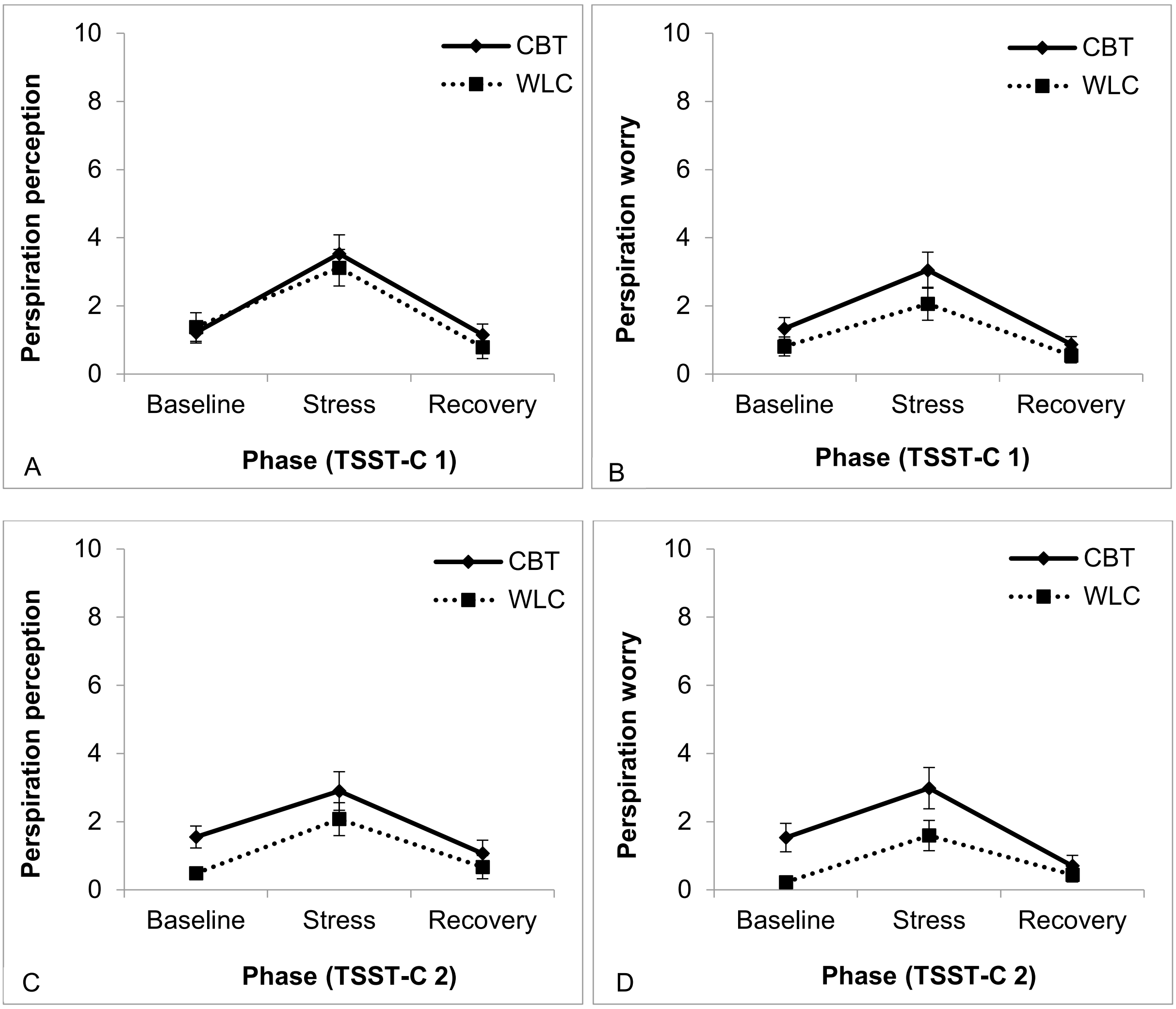

Figure S4. Subjective perception of and worry about perspiration after all phases of the first (A, B) and second (C, D) Trier Social Stress Test for Children (TSST-C), comparing the cognitive behavior therapy (CBT) and waitlist control (WLC) groups.

For subjective perception of blushing after treatment, the ANOVA showed a significant main effect of phase, Wilk's $\lambda = .566$, $F_{(4,49)} = 9.38$, $p < .001$, $\eta_p^2 = .434$. All other Fs < 2.06, ps > .100 (see Figure S5).

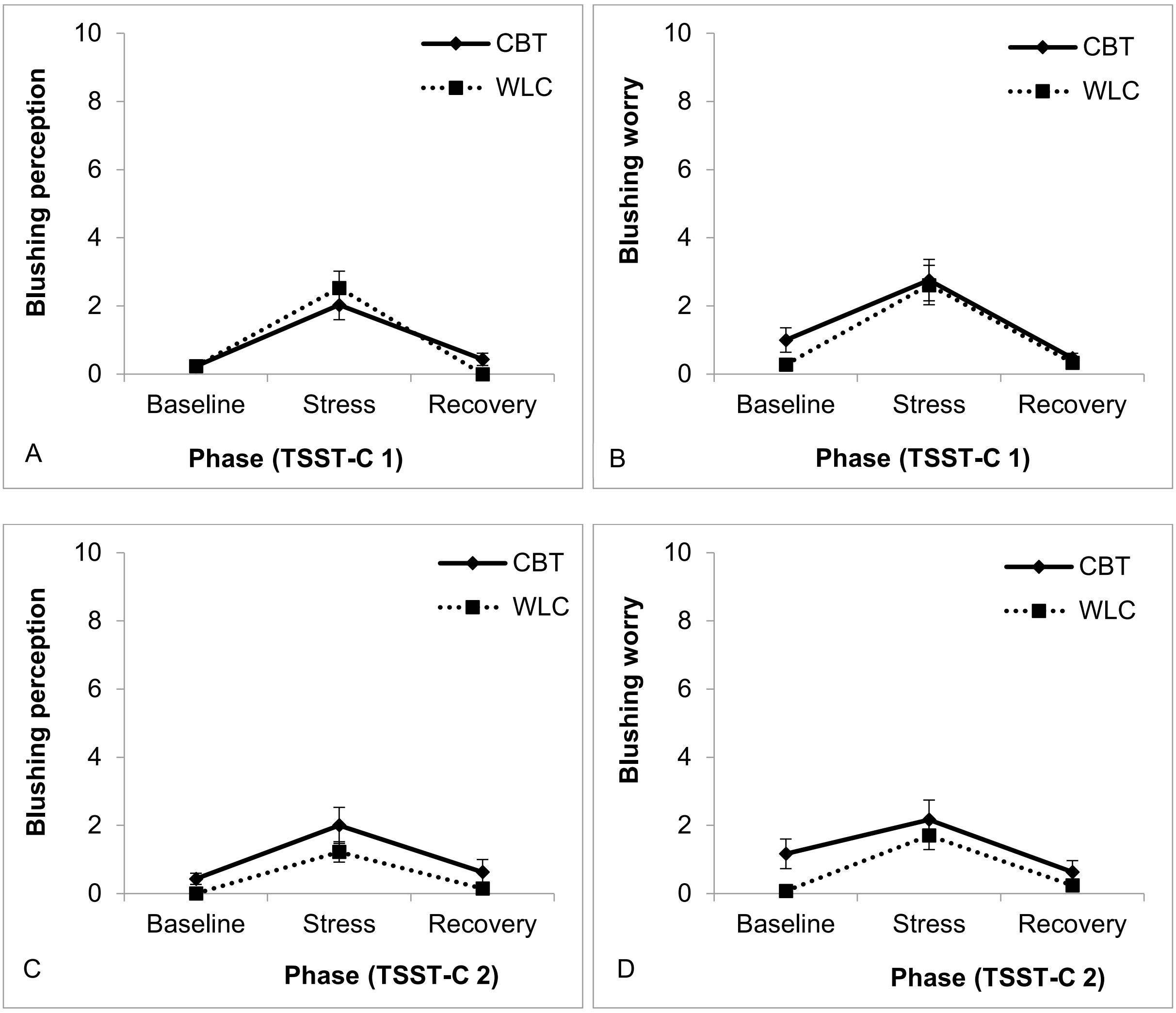

Figure S5. Subjective perception of and worry about blushing after all phases of the first (A, B) and second (C, D) Trier Social Stress Test for Children (TSST-C), comparing the cognitive behavior therapy (CBT) and waitlist control (WLC) groups.

For subjective perception of trembling after treatment, the ANOVA showed significant main effects of session, Wilk's $\lambda = .244$, $F_{(2,51)} = 3.56$, $p = .036$, $\eta_p^2 = .122$, and phase, Wilk's $\lambda = .459$, $F_{(4,49)} = 14.41$, $p < .001$, $\eta_p^2 = .541$, and a significant interaction effect for Phase × Group, Wilk's $\lambda = .787$, $F_{(4,49)} = 3.32$, $p = .018$, $\eta_p^2 = .213$. All other *F*s < 1.31, *p*s > .279. Post hoc *t* tests for independent samples showed a significantly higher worry about trembling at TSST-C 2 in the CBT group after the baseline phase, $t_{(45.7)} = 2.05$, $p = .046$, $d = 0.54$ (see Figure S6). All other *t*s < 1.41, *p*s > .163. After the baseline phase posttreatment,

children in the CBT group reported higher worry about the visibility of trembling than children in the WLC group.

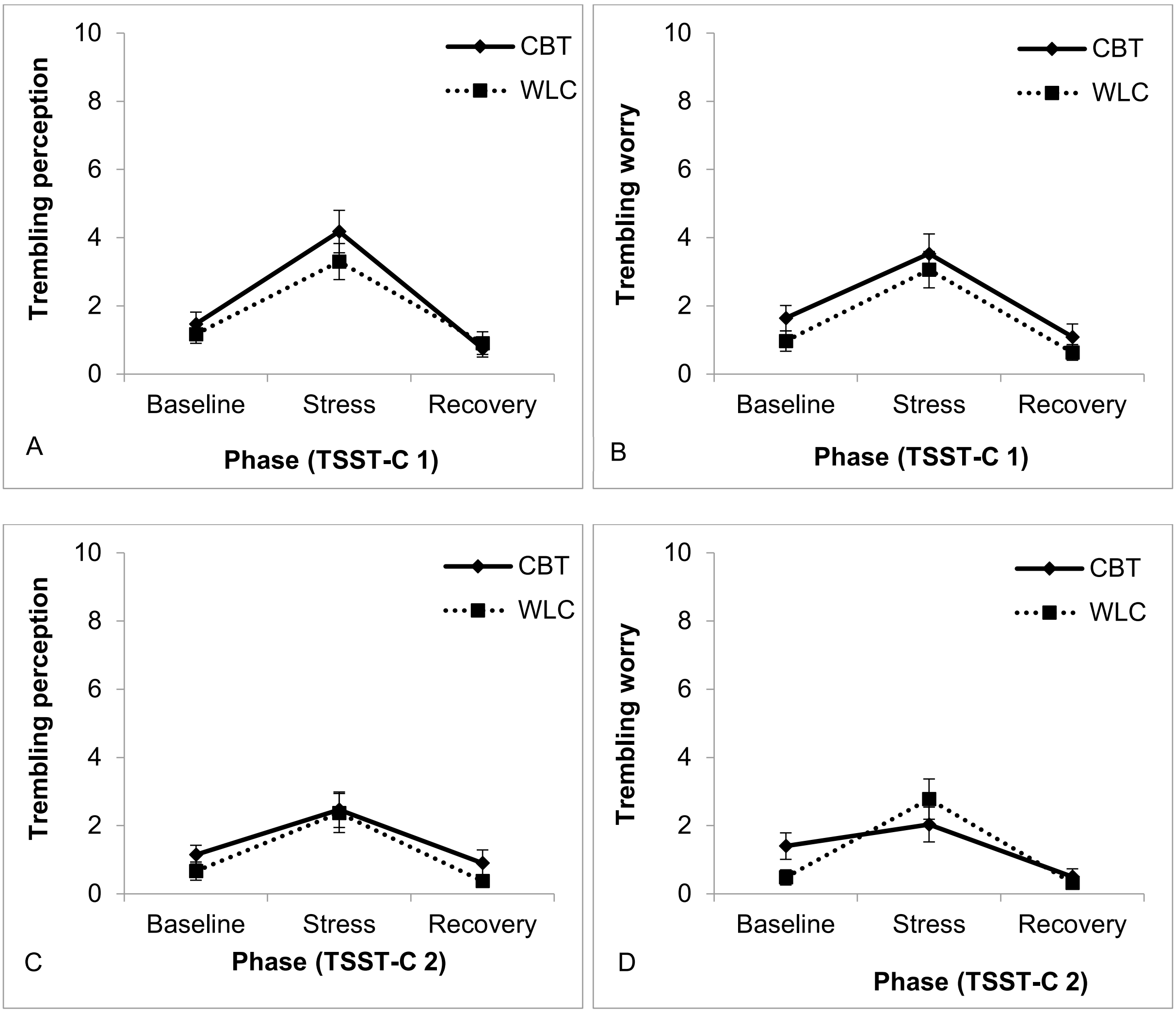

Figure S6. Subjective perception of and worry about trembling after all phases of the first (A, B) and second (C, D) Trier Social Stress Test for Children (TSST-C), comparing the cognitive behavior therapy (CBT) and waitlist control (WLC) groups.
